# Acceptability of HIV self-testing among sexual health service providers and Two-Spirit, gay, bisexual, and queer men in Ontario, Canada

**DOI:** 10.1093/heapro/daag045

**Published:** 2026-04-02

**Authors:** Heeho Ryu, Mackenzie Stewart, Joshun Dulai, Oralia Gómez-Ramírez, Catherine Worthington, Mark Gilbert, Daniel Grace

**Affiliations:** Dalla Lana School of Public Health, University of Toronto, 155 College Street, 5th Floor, Room 510, Toronto, Ontario M5T 3M7, Canada; Dalla Lana School of Public Health, University of Toronto, 155 College Street, 5th Floor, Room 510, Toronto, Ontario M5T 3M7, Canada; Dalla Lana School of Public Health, University of Toronto, 155 College Street, 5th Floor, Room 510, Toronto, Ontario M5T 3M7, Canada; BC Centre for Disease Control, 655 W 12th Ave, Vancouver, British Columbia V5Z 4R4, Canada; School of Public Health and Social Policy, University of Victoria, 3800 Finnerty Road, Victoria, British Columbia V8P 5C2, Canada; BC Centre for Disease Control, 655 W 12th Ave, Vancouver, British Columbia V5Z 4R4, Canada; School of Population and Public Health, University of British Columbia, 2206 East Mall, Vancouver, British Columbia V6T 1Z3, Canada; Dalla Lana School of Public Health, University of Toronto, 155 College Street, 5th Floor, Room 510, Toronto, Ontario M5T 3M7, Canada

**Keywords:** HIV self-testing, STBBI prevention, 2S/GBTQ + health, sexual health systems, Canada

## Abstract

In 2020, Health Canada approved the INSTI human immunodeficiency virus (HIV) self-test. Adoption and distribution of alternative HIV testing interventions, like self-testing, are essential in meeting the United Nations 95-95-95 goals. We explored the acceptability of HIV self-testing among sexual health service providers and Two-Spirit, gay, bisexual, and queer men (2SGBQM). Between 2020 and 2021, peer researchers conducted virtual focus groups (13) and interviews (18) with providers (*n* = 18) and 2SGBQM (*n* = 38) across Ontario, Canada, and analysed data using community-based participatory research approach and reflexive thematic analysis. HIV self-testing was highly acceptable among both providers and 2SGBQM. Both groups identified ‘increased access to HIV testing’ as a benefit. Providers identified ‘client empowerment’ and ‘reduced workload for providers’ as other perceived benefits. 2SGBQM highlighted ‘convenience’ as a key benefit and rationale for self-testing, though some expressed concerns and hesitance due to ‘fear of needles/blood’ and ‘perceptions of lower accuracy and reliability’ of self-test results. Providers and 2SGBQM referred to the ‘potential for missed connections to care’, and ‘self-harm’ with positive test results as additional concerns for self-testing. Both groups suggested that first-time or inexperienced testers should be tested in-clinic, compared with experienced or regular testers who may benefit from self-testing. Participants expressed that HIV self-testing should be widely available for free, or a modest fee up to $20 CAD. Providers and 2SGBQM both found HIV self-testing highly acceptable, particularly when self-administered by experienced testers. Clinic-based testing remains important, especially for first-time testers and 2SGBQM who have concerns or hesitance regarding self-testing.

Contribution to Health Promotion

- Highlights the need for innovative HIV testing using individual and structural perspectives on self-testing.
- The results and existing literature can support decision-making processes related to the design and implementation of self-testing promotion in public health institutions.
- Highlights the gaps in health policy regarding HIV testing by supporting why further funds should be allocated by the Canadian government after the discontinuation of support to self-testing programmes in 2024.
- Novel contributions aligned with current literature that provide a sustained look at HIV self-testing across different stages of implementation and support further calls to action that are applicable outside of Canada.

## Introduction

The Public Health Agency of Canada estimates that in 2020, 62,790 people were living with human immunodeficiency virus (HIV) in Canada, and that 10% of this population was unaware of their sero-positivity status (PHAC 2022a). HIV disproportionately affects members of intersecting population groups such as gay, bisexual, and other men who have sex with men; African, Caribbean, and Black (ACB) communities; people who inject drugs (PWID); and Indigenous Peoples (i.e. First Nations, Métis, and Inuit communities) (PHAC 2015, 2020). Of the 1520 new HIV infections reported in Canada in 2020, nearly half (44%) of the diagnoses were among gay, bisexual, and other men who have sex with men and one-fifth (18%) among Indigenous Peoples, despite the two groups representing just 4% and 5% of the adult population, respectively (PHAC 2022a, Sorge *et al*. 2023, Statistics Canada 2024). Similarly, gay, bisexual, and other men who have sex with men represented the majority (61%) of first-time HIV diagnoses in Ontario in 2021, followed by ACB individuals (25%), PWID (15%), and Indigenous Peoples (6%) (OHESI 2022).

This disparate burden of HIV is due in part to additional social and structural barriers these groups face in accessing testing services, including: stigma and judgment from service providers based on ethnicity, sexual orientation, or substance use; providers’ lack of knowledge regarding community-specific health needs; fear of stigma and isolation due to breach of anonymity and confidentiality; and geographic barriers in rural and remote regions, especially on Indigenous reserves (Negin *et al*. 2015, Jongbloed *et al*. 2019, Laprise *et al*. 2021, Etowa *et al*. 2022, Stewart *et al*. 2022, Dulai *et al*. 2023, 2024, Hassan *et al*. 2025). Alternative testing modalities like HIV self-testing could help address these barriers, reducing the burden of HIV and improving sexual health outcomes for priority population groups across Canada.

HIV self-testing interventions use either saliva or finger-prick blood samples, and both types have been found to be acceptable and feasible (i.e. users can follow instructions, collect samples, administer a rapid test, and interpret results on their own) among diverse populations across Sub-Saharan Africa, East Asia, North America, and Oceania (Pant Pai *et al*. 2014, 2018a, Witzel *et al*. 2020, Jamil *et al*. 2021, O'Byrne *et al*. 2021a, 2021b, 2023a, 2023b). Studies have also demonstrated that both types of HIV self-testing interventions are effective in removing barriers and increasing opportunities for HIV testing, diagnosis, and treatment (Pant Pai *et al*. 2014, 2018a, Witzel *et al*. 2020, Jamil *et al*. 2021, O'Byrne *et al*. 2021a, 2021b, 2023a, 2023b). Hence, HIV self-testing is recommended by the World Health Organization (WHO) as a convenient, confidential, and accurate testing modality that can benefit priority population groups and reach individuals who are missed in traditional clinic-based testing models (WHO 2019).

The first HIV self-test in Canada, INSTI, was approved by Health Canada in November 2020 (PHAC 2020). INSTI is a finger-prick self-test manufactured by bioLytical Laboratories with reported sensitivity (probability of a positive test result if true positive) of 99.8% and specificity (probability of a negative test result if true negative) of 99.5% in detecting HIV (Galli *et al*. 2021, bioLytical Labratories 2025a). In March 2023, Health Canada further approved the licence of a dual HIV-syphilis rapid self-test, also produced by bioLytical Laboratories (Unity Health Toronto 2023), with reported sensitivity and specificity of 99.8% and 99.5%, respectively for HIV, and 99.5% and 98% for syphilis (Singh *et al*. 2023, bioLytical Labratories 2025b).

HIV self-testing can play a significant role in meeting the goals of the Pan-Canadian sexually transmitted and blood-borne infections (STBBI) Framework for Action (e.g. increasing the availability of and access to effective and innovative testing modalities), and the UNAIDS 95-95-95 targets to diagnose 95% of all people living with HIV, provide antiretroviral therapy (ART) to 95% of the diagnosed population, and achieve viral suppression among 95% of those receiving treatment by 2030 (UNAIDS 2014, PHAC 2018). Recognizing this, the Government of Canada made a one-time commitment of $8 million CAD for the distribution of HIV self-testing kits across Canada in August 2022 (PHAC 2022b). HIV self-testing kits were made available through various channels: private sales through bioLytical Laboratories (bioLytical Laboratories 2025c) or pharmacies (HIV Testing Ontario 2020, The Village Pharmacy 2025); research trials such as ‘GetaKit’ (https://getakit.ca/) (O'Byrne *et al*. 2021a, 2021b, 2023a, 2023b) and ‘I’m Ready’ (https://www.reachnexus.ca/) (I'm Ready 2021, 2022); or community-based programmes such as ‘Test@Home’, ‘TestNow’, and ‘Medicine Bundle’ from the Community-Based Research Centre (https://www.cbrc.net/projects_and_initiatives) (CBRC 2025a, 2025b, 2025c), as well as Our Healthbox from REACH NEXUS (https://www.ourhealthbox.ca/) (MAP 2025, Our HealthBox 2025). However, this funding from the Government of Canada ended on 31 March 2024 and no further commitments for the distribution of HIV self-testing kits have been made since (CBC News 2024, Malone 2024).

### Study aim

Given that HIV self-testing has the potential to help address HIV testing barriers and disparities faced by priority population groups, our objective was to explore the acceptability of HIV self-testing and factors influencing its adoption (i.e. perceived benefits and concerns) among sexual health service providers and service users who self-identify as Two-Spirit, gay, bisexual, queer, and other men who have sex with men (2SGBQM) in Ontario, Canada.

## Materials and methods

### Study design

Between June 2020 and December 2021, we conducted 60–90-min semi-structured virtual focus groups and interviews with 18 sexual health service providers and 38 2SGBQM service users in Ontario, Canada. This analysis is part of a community-based participatory research (CBPR) study investigating how access to sexual health testing could be improved through the implementation of alternative testing modalities such as HIV self-testing and online-based testing (Grace *et al*. 2022). CBPR is a research approach ensuring full and equal participation of community members who are directly impacted by the issue being studied, through co-learning and sharing power over the research process (Northridge *et al*. 2007, Rhodes *et al*. 2010). Following CBPR principles, four peer researchers self-identifying as members of the 2SGBQM community with multiple intersecting identities (as *trans*, non-binary, Indigenous, Black, and/or person of colour) conducted recruitment, data collection, analysis, and knowledge translation. A community advisory board (CAB) comprising 2SGBQM with professional or personal experience and knowledge of sexual and gender minority health was consulted throughout the study to provide feedback and help direct the research. This study was approved by the University of Toronto Research Ethics Board.

### Recruitment

Recruitment and data collection took place over three phases. Between June 2020 and February 2021, 31 2SGBQM (phase 1) and 18 providers (phase 2) were recruited, respectively, and participated in either focus groups or interviews. Following consultations from the CAB, we identified an underrepresentation of Indigenous and Black 2SGBQM in our sample and conducted a third phase (September-December 2021) of recruitment and data collection, recruiting seven Indigenous and Black 2SGBQM to participate in focus groups or interviews.

Approval of Canada’s first HIV self-testing device coincided with phase 2 of recruitment and data collection. Phase 1 took place prior to the approval, and phase 3 was conducted a year after the approval. Purposive and snowball sampling methods were used to recruit participants (Morse 2007). Purposive sampling ensured the recruitment of participants with relevant professional experience providing sexual health care or lived experience of accessing sexual health testing services as 2SGBQM. Recruitment posters were distributed on social media platforms (i.e. Facebook, Instagram, and Twitter), as well as by email to acquired immunodeficiency syndrome (AIDS) service organizations, pride organizations, community-based organizations, community health centres, public health units, and sexual health clinics. Snowball sampling in the form of peer referral helped to reach a wider network of providers and 2SGBQM; participants were encouraged to share the opportunity to participate in our research within their professional or personal networks. An eligibility screener on SurveyMonkey was completed by interested providers and 2SGBQM, and those who met the study criteria were invited to participate. Eligibility criteria for 2SGBQM participants included: self-identifying as 2SGBQM; being 18 years of age or older at the time of participation; and having tested for sexually transmitted infections (STIs) or HIV within the past 12 months. For providers, the eligibility criteria included: experience working directly with sexual health service users, and currently working in Ontario.

### Data collection

Participants who provided their written consent and completed a sociodemographic survey on Qualtrics were scheduled for a focus group (comprising two to six participants) or interview based on their availability and comfort level. Due to the onset of the COVID-19 pandemic, data collection took place online using Microsoft Teams and Zoom conferencing software. Each session was audio and video recorded and stored on a secure online drive. Audio recordings were transcribed verbatim and participants were assigned a pseudonym to ensure anonymity. We adopted the principle of theoretical saturation from grounded theory as a pragmatic criterion to conclude data collection when no new insights or perspectives related to the study objectives were present in the data collected from additional focus groups or interviews (Charmaz and Belgrave 2012), allowing the size of our dataset to be determined by the richness of the data rather than quotas. 2SGBQM participants were each compensated $30 CAD for their participation.

Focus group and interview guides were developed by the principal investigator and research coordinator and subsequently reviewed by the peer researchers. Both providers and 2SGBQM were asked about their thoughts and impressions of finger-prick HIV self-tests, the perceived benefits and concerns with this testing modality, and how much a self-test should cost to purchase. Providers were specifically asked about the potential impact that HIV self-testing may have on their clients, workplaces, and the provincial public health landscape. 2SGBQM were asked about their interest in and likelihood of using HIV self-tests. For the full focus group and interview guides, consult Grace *et al*. (2022, supplemental file 1) for the provider guide, and Stewart *et al*. (2022, supplemental file 1) for the 2SGBQM service user guide.

### Data analysis

Transcripts were coded using reflexive thematic analysis (RTA) techniques as originally described (Braun and Clarke 2006) and subsequently elaborated by Braun *et al*. (2019) and Braun and Clarke (2023), aided by NVivo 12 software. RTA is an analytic approach that acknowledges and centres the active involvement of researchers’ subjectivities and biases throughout the interpretation and analysis of qualitative data, employing an iterative and open coding process to identify ‘themes’ or patterns of shared meaning (Braun and Clarke 2006, 2023, Braun *et al*. 2019). Rather than using predetermined themes or fixed coding frameworks, RTA allows for an organic evolution of codes and themes through stages of familiarization, generating initial codes, searching for themes, reviewing themes, and defining and naming themes (Braun and Clarke 2006, 2023, Braun *et al*. 2019). RTA complements CBPR principles and our research approach in centring 2SGBQM peer researchers as co-producers of knowledge, and their lived experiences as critical lenses for data interpretation and shared meaning-making.

Peer researchers were familiarized with the data during the transcription phase through a thorough review of audio and video recordings from focus groups and interviews. After independently analysing and developing initial codes for each transcript, peer researchers discussed emerging patterns and common themes across the data. The principal investigator and research coordinator were consulted to further refine and develop codes and themes, reflexively engaging with their own positionalities as HIV researchers and members of the 2SGBQM community. A coding scheme was created, and peer researchers reviewed and recoded each transcript using the new framework. The data was subsequently refined and reorganized into the developed themes and subthemes throughout the analysis phase.

## Results

### Participant characteristics

In total, we conducted 10 focus groups and 7 interviews with 2SGBQM service users (*n* = 38) and 3 focus groups and 11 interviews with sexual health service providers (*n* = 18). 2SGBQM participants were 32 years old on average, around half identified as White (47%), and the majority identified as gay (68%) and cisgender (63%). Most completed some form of postsecondary education (85%), and had accessed STI (79%) or HIV (77%) testing within 6 months prior to participation. Table 1 provides demographic information for the 2SGBQM participants.

**Table 1:** 2SGBQM service user demographics.

	*N* = 38
**Age**	
18–24	6 (16%)
25–34	22 (58%)
35–44	5 (13%)
45+	5 (13%)
**Race/ethnicity** ^a^	
White	18 (47%)
African/Caribbean/Black	6 (16%)
Indigenous/First Nations	4 (11%)
Southeast Asian	3 (8%)
Latin American	3 (8%)
Middle Eastern	2 (5%)
East Asian	1 (3%)
South Asian	1 (3%)
Indian-Caribbean	1 (3%)
**Sexual orientation** ^a^	
Gay	26 (68%)
Bisexual/pansexual	8 (21%)
Queer	4 (11%)
Asexual	1 (3%)
Demisexual	1 (3%)
Straight^b^	1 (3%)
Two-Spirit^c^	1 (3%)
Questioning	1 (3%)
**Gender** ^a^	
Cisgender	24 (63%)
Transgender	8 (21%)
Non-binary	4 (11%)
Prefer not to answer	3 (8%)
Two-Spirit^c^	2 (5%)
**Highest level of education**	
Did not complete high school	2 (5%)
High school or equivalent	4 (11%)
Postsecondary certificate or diploma	9 (24%)
Bachelor’s degree	14 (37%)
Above a bachelor’s degree	9 (24%)
**Most recent STI test**	
≤ 3 months ago	19 (50%)
4–6 months ago	11 (29%)
7–12 months ago	4 (11%)
>12 months ago	3 (8%)
Never	1 (3%)
**Most recent HIV test**	
≤ 3 months ago	15 (40%)
4–6 months ago	14 (37%)
7–12 months ago	4 (11%)
>12 months ago	5 (13%)

^a^Totals may exceed 100% as categories are not mutually exclusive.

^b^One participant identified as ‘straight’ for their sexual orientation but ‘Two-Spirit’ for gender identity, satisfying requirements for participation in the study.

^c^In total, three distinct participants identified as ‘Two-Spirit’; the term Two-Spirit encompasses diverse sexual orientations, gender identities, roles, and expressions that vary across different Indigenous Peoples and cultures.

The occupations of our provider participants varied, with the majority working as either frontline workers at community-based or HIV/AIDS service organizations (39%) or sexual health nurses (22%), followed by physicians (11%), public health workers (11%), clinic counsellors/HIV testing professionals (11%), and manager of sexual health nurses (6%). Half (50%) had over 5 years of experience in their roles, with two (11%) having <1 year of experience. Most served large urban regions (94%), and priority population groups including 2SGBQM (83%) and PWID (44%). Table 2 presents demographic information of the provider participants.

**Table 2:** Sexual health service provider demographics.

	*N* = 18
**Occupation**	
Frontline worker	7 (39%)
Sexual health nurse	4 (22%)
Physician	2 (11%)
Public health worker	2 (11%)
Clinic counsellor/HIV testing professional	2 (11%)
Manager of sexual health nurses	1 (6%)
**Years of experience**	
>5 years	9 (50%)
1–5 years	7 (39%)
<1 year	2 (11%)
**Area served**	
Greater Toronto Area	7 (39%)
Ottawa	2 (11%)
Other large urban area (>100 000)^a^	8 (44%)
Rural community (1000 > 29 000)^b^	1 (6%)
**Populations served** ^c^	
2SGBQM	15 (83%)
PWID	8 (44%)
Trans/gender non-conforming individuals	7 (39%)
Sex workers	5 (28%)
Youth	2 (11%)
Adults between 18 and 60	1 (6%)
Immigrants/newcomers	1 (6%)

^a^Include Barrie, Guelph, Kingston, Kitchener-Waterloo, London, and Richmond Hill.

^b^Includes Muskoka region.

^c^Totals may exceed 100% as categories are not mutually exclusive.

### Findings

Support for the adoption and distribution of HIV self-tests using finger-prick blood samples, like INSTI, was very high among both sexual health service providers and 2SGBQM service users. Though only one 2SGBQM participant had heard of HIV self-testing prior to participation, about a third of 2SGBQM reported that they would regularly access HIV self-testing once it became available, while around half expressed that they would access HIV self-testing but continue regular testing through their existing service provider. Other 2SBGQM explained that accessing HIV self-testing would necessitate weighing its perceived benefits and concerns within the context of their sexual health risks, wants, and needs. Both providers and 2SGBQM suggested that HIV self-tests should be available for free. When asked to assign a reasonable cost for purchasing a self-testing kit, they agreed that $20 CAD or less felt the most appropriate since pregnancy tests, a widely available point-of-care test in Ontario, cost around $20 CAD or less. Prices above $20 CAD were considered inaccessible, in line with previous Canadian studies which also identified $20 CAD as the most appropriate price for HIV self-tests among providers and sexual health service users (Pant Pai *et al*. 2014, 2018a, 2018b). Table 3 summarizes participant’s perceived benefits and concerns with HIV self-testing.

**Table 3:** Perceived benefits and concerns with HIV self-testing.

	2SGBQM service users (*n* = 38)	Sexual health service providers (*n* = 18)
Benefits	**Convenience** e.g. ‘[HIV self] testing kits that can give you immediate results at home in general are appealing to me […] I really value convenience and immediate results’	**Convenience** e.g. ‘the process [of testing] itself usually doesn’t need to take too long, but sometimes you’ve been there [at the clinic] for an hour or two’
	**Increased access** e.g. ‘I have gone to [walk-in clinics] for testing, and it’s amplified my anxiety too much when I’ve got there or I’ve felt that I’ve been judged, and so I’ve turned around and left’	**Increased access** e.g. ‘I think what [HIV self-testing] would do is possibly be a gateway to accessing services for some people [who] just don’t want to see a healthcare provider’
	**Client empowerment** e.g. ‘I would prefer [to self-test at] home […] being able to control and [test] on my own pace and rate’	**Client empowerment** e.g. ‘It’s a bit of a principle of empowerment. Like [with HIV self-testing] people have control of their own sexual health’
		**Reduced workload** e.g. ‘[HIV self-testing] just provides another option, especially during COVID times, of accessible HIV testing […] a less supervised approach would be one that we take just for more ease of access, and less administrative burden for us’
Concerns	**Fear of needles/blood** e.g. ‘For me, I don’t like finger pricks, I rather [a provider] take my blood, that is just a weird personal preference. So I personally wouldn’t use [HIV self-testing]’	**Fear of needles/blood** e.g. ‘I think people are needle-phobic […] that may be a barrier for people. But if it’s a saliva test […] I think there would be greater uptake [of self-testing]’
	**Accuracy and reliability** e.g. ‘I guess [uptake] would depend on the efficacy of the [HIV self] test; I don’t know whether there’s a difference between getting your blood drawn [at the clinic] and having your blood tested [with] the pinprick’	
	**Missed connections to care, self-harm/suicide ideation** e.g. ‘There’s still like a stigma with HIV and there’s still a high suicide rate with HIV folks. And there I feel like there’s a risk if someone does test positive at home and they don’t have support’	**Missed connections to care, self-harm/suicide ideation** e.g. ‘People were very concerned about home pregnancy tests when they came on the market and they’re fine […] I don’t have actual huge concerns that people are going to do their tests wrong, or they’re going to misuse them’

### Perceived benefits of human immunodeficiency virus self-testing

We identified four overarching themes regarding the perceived benefits of HIV self-testing among providers and 2SGBQM: ‘(i) convenience as a key benefit and factor in the uptake of HIV self-testing; (ii) increased access to HIV testing through removal of social and structural barriers to testing; (iii) client empowerment and self-autonomy in sexual health care; and (4) reduced workload for providers and increased capacities for comprehensive sexual health services’.

#### Convenience as a key benefit and factor in the uptake of human immunodeficiency virus self-testing

2SGBQM highlighted temporal convenience as the greatest perceived benefit of HIV self-testing. They reported long travel and wait times, inconvenient clinic hours, and lack of available appointments as major barriers and deterrents to seeking HIV testing, and providers also echoed these sentiments and identified inconvenience as one of the greatest barriers for their clients seeking HIV testing. Isaac, an HIV testing professional illustrated this point:

Having to wait for a long time for something that, in theory, could take 10 minutes […] the process [of testing] itself usually doesn’t need to take too long, but sometimes you’ve been there [at the clinic] for an hour or two. (Service Provider Interview)

In contrast, both 2SGBQM and providers believed that HIV self-testing would be much less time consuming than traditional clinic-based testing. 2SGBQM shared that convenience—the total time and effort required to receive testing—was the greatest factor influencing their decision to test with a particular testing modality, and valued the potential for HIV self-testing to increase convenience. Ian (20s, *trans* and non-binary) put it like this:

[HIV self] testing kits that can give you immediate results at home in general are appealing to me […] I really value convenience and immediate results. So I would find [HIV self-testing] appealing. (Service User Interview)

Other 2SGBQM also shared Ian’s perspective, and further identified recent exposures to sexual health risks or HIV as situations where the convenience of HIV self-testing would be particularly appealing. Most 2SGBQM expressed that they would use or consider using HIV self-tests in the future, especially if they were unable to access clinic-based testing in a timely manner.

### Increased access to human immunodeficiency virus testing through removal of social and structural barriers to testing

Both 2SGBQM and providers believed that HIV self-testing would increase access by removing the social and structural barriers associated with traditional clinic-based testing. These barriers included: lack of anonymity and confidentiality; fear and anxiety; and identity-based stigma from providers. To illustrate this point, Bailey (30s, *cis* man) described his own experience of foregoing HIV testing due to anxiety and perceived judgment from providers:

I have gone to [walk-in clinics] for testing, and it’s amplified my anxiety too much when I’ve got there or I’ve felt that I’ve been judged, and so I’ve turned around and left. (Service User Focus Group)

Similarly, a director of a local public health unit also shared his concerns for those living in rural and remote areas who often forego HIV testing due to fear from a lack of anonymity and confidentiality within their local clinics:

They’re a somewhat more well-known member of the community, and they don’t really want to be seen at a sexual health clinic where there are people that might know them […] I would say probably more conservative communities; that anonymity [from HIV self-testing] would be beneficial to them (Ford, Service Provider Interview)

Moreover, both 2SGBQM and providers suggested that HIV self-testing could be a ‘gateway’ or entry point for first-time and infrequent testers’ to start their sexual healthcare journey. Eden, a public health worker explained:

I think what [HIV self-testing] would do is possibly be a gateway to accessing services for some people [who] just don’t want to see a healthcare provider, and I think you’re going to see those people doing the self-test. (Service Provider Interview)

Overall, both 2SGBQM and providers agreed that alternative testing modalities such as HIV self-testing that is anonymous and can eliminate stigma and anxiety could increase access to HIV testing for those who have been unable or unwilling to get tested due social and structural barriers to clinic-based testing.

### Client empowerment and self-autonomy in sexual health care

Client empowerment was identified by providers as another potential benefit of HIV self-testing, and they discussed how self-testing could enable individuals to take control of their own sexual health without intervention from providers. A director of a local public health unit drew on the concept of patient agency, explaining:

It’s a bit of a principle of empowerment. Like [with HIV self-testing] people have control of their own health […] rather than having a healthcare provider tell you what you need to do all the time. (Ford, Service Provider Interview)

Ford’s belief that HIV self-testing could empower individuals to control their own sexual health was corroborated by 2SGBQM like Jack (20s, *cis* man), who shared that he would prefer to use HIV self-testing specifically ‘to control and [test] on my own pace and rate’ (Service User Focus Group).

In another focus group, Anthony (20s, *cis* man) who was living with HIV suggested that if HIV self-testing had been available at the time of his diagnosis, it would have given him more agency within his HIV journey:

Not having to deal with again someone judging you, because now you’re getting tested for HIV […] if [HIV self-testing] was around back when I was getting diagnosed and stuff, I would have gone that route. (Service User Focus Group)

Ultimately, providers highly valued the profound impact that HIV self-testing could have on enhancing client autonomy, allowing individuals to navigate their own sexual health journey with greater independence and confidence, free from judgement or external pressures. The personal experiences and preferences expressed by service users like Jack and Anthony deeply resonate with providers’ sentiment that HIV self-testing has the potential to foster a more empowering healthcare environment.

### Reduced workload for providers and increased capacities for comprehensive sexual health services

Providers highlighted the potential to reduce their workload, time, and resources spent on HIV testing services as another foreseeable benefit of HIV self-testing. For instance, providers discussed how HIV self-testing could free up organizational capacity by having clients self-administer tests at home or the clinic. Ford, a director of a public health unit explained:

I think [HIV self-testing] just provides another option, especially during COVID times, of accessible HIV testing […] a less supervised approach would be one that we take just for more ease of access, and less administrative burden for us. (Service Provider Interview)

Providers believed that in turn, reduced workloads and resources saved would allow them to improve clients’ access to and experience of clinic-based testing, while also improving the quality and availability of other services and programmes. Kendall, a front-line worker, illustrated the challenges he faces daily in balancing his work between sexual health testing and other clinic-based services:

Balancing our demand for appointments versus our capacity as a pretty small clinical team has been an ongoing challenge for us […] we don’t just do testing and sexual health stuff, we also do primary care, we do mental health care. (Service Provider Interview)

From the perspective of the providers, HIV self-testing presented a significant opportunity to better manage their workload and alleviate existing strains on sexual health clinics. They believed that enabling clients to self-test for HIV may allow service providers to enhance their quality of care and ensure that clients have greater access to a more comprehensive range of sexual and other healthcare services and programmes.

### Potential concerns with human immunodeficiency virus self-testing

While recognizing the potential benefits of HIV self-testing, 2SGBQM and providers also shared their concerns regarding the availability and uptake of HIV self-testing. We identified three overarching themes: ‘(i) fear of needles/blood as a key barrier in the uptake of HIV self-testing; (ii) perception of lower accuracy and reliability of HIV self-testing results; and (iii) potential for missed connections to care, self-harm, and suicide ideation’.

### Fear of needles/blood as a key barrier in the uptake human immunodeficiency virus self-testing

Some 2SGBQM highlighted their fear of needles/blood as the main reason for their hesitancy in accessing HIV self-testing. They expressed that it would be challenging to self-administer a pin-prick needle to draw droplets of blood, and that they would prefer to get tested in-person with the help of a provider. Sean (20s, *trans* and non-binary) explained:

For me, I don’t like finger pricks, I rather [a provider] take my blood, that is just a weird personal preference. So I personally wouldn’t use [HIV self-testing]. (Service User Focus Group)

Providers similarly identified the fear of needles/blood as a key barrier in the uptake of HIV self-testing among some of their clients.

I think people are needle-phobic, so if people need to stab themselves with a lance, that may be a barrier for people. (Ford, Service Provider Interview)

Nonetheless, both 2SGBQM and providers acknowledged that HIV self-testing would still be a beneficial testing method for individuals who are comfortable with the self-collection of blood samples. Some of the 2SGBQM who indicated discomfort with needles/blood even shared that they may opt for HIV self-testing in situations where there is an urgent need to test for HIV.

### Perception of lower accuracy and reliability of human immunodeficiency virus self-testing results

Among some 2SGBQM, there was a perception that HIV self-testing may ‘not [be] as accurate, or confirmatory as the [clinic-based] blood test’ (Emile, 20s, *cis* man, Service User Focus Group). This impression of lower accuracy and reliability posed as a deterrent in the uptake of HIV self-testing, as it presented HIV self-testing as either redundant or misleading.

The first tension was that regardless of the result of an HIV self-test, self-testers may feel the need to seek confirmatory testing with a provider, creating redundancy. 2SGBQM who held this belief expressed that they would rather get tested once at the clinic using the blood-draw test—which they perceived to be more definitive—compared with getting tested twice with an HIV self-test followed by another confirmatory test. Within this rationale, the time and resources spent on accessing HIV self-testing felt unnecessary and wasteful when clinic-based testing was available for free as a more reliable option. Bernard (50s, *cis* man) shared his perspective on this matter:

I guess [uptake] would depend on the efficacy of the [HIV self] test; I don’t know whether there’s a difference between getting your blood drawn [at the clinic] and having your blood tested [with] the pinprick […] how definitive you want that test to be and whether you have time to get a second test. (Service User Focus Group)

Simultaneously, some 2SGBQM were concerned that if HIV self-testing was not as accurate as the blood-draw test, it may result in false negatives or positives which would not only mislead and harm individual testers, but also have repercussions for their sexual partners. In the case of false negatives, 2SGBQM were worried that it would lead to individuals not being aware of their sero-positivity status, not seeking treatment, and unknowingly transmitting HIV to their sexual partners. 2SGBQM were especially concerned for those who are less informed about HIV or are less experienced with HIV testing. Cameron (20s, *cis* man) described this in detail:

My concern is that, not everybody is well-informed […] they took the HIV [self-testing] kit and they test negative, they have the self-reassurance that, ‘oh ok, I’m fine. I’m negative.’ But actually, it takes time for the antibodies to show up. (Service User Focus Group)

Alternatively, in the case of false positives 2SGBQM were concerned that some individuals would experience unwarranted and significant emotional distress. They explained that first-time or less-informed self-testers may believe their positive self-test result to be the definitive diagnosis, when in reality confirmatory blood-draw testing is necessary to corroborate that result. 2SGBQM were further concerned that some individuals may hold HIV stigma while not being aware of the advances in HIV treatment and care, or that ‘the face of HIV has changed dramatically’ (Allard, 30s, *cis* man, Service User Focus Group). Here, Allard is referring to how regular monitoring and treatment through ART empower individuals with positive serostatus to continue living healthy and fulfilling lives. Given such concerns, 2SGBQM emphasized that having sexual health professionals available before accessing HIV self-testing would be important and even necessary to help self-testers learn more about HIV, HIV testing, treatment options, and additional resources that are available in case of a positive result.

### Potential for missed connections to care, self-harm, and suicide ideation

Both 2SGBQM and providers further identified the potential for missed connections to care such as counselling, education, and treatment as an additional concern with HIV self-testing. Providers shared their experience and highlighted HIV stigma as a significant barrier for clients in seeking the next steps in their HIV care continuum. Heidi, a manager of sexual health nurses, shared her perspective:

[Some clients are] like, ‘no, I don’t want the bloodwork’ and they just leave. Because I think they suspect [sero-positivity and] aren’t ready for the next step […] we have people who reach out and say, ‘you know what, I was tested a year ago, and I was positive, and I didn’t do anything, and I’m ready now’. (Service Provider Interview)

In this context, both 2SGBQM and providers agreed that easy and timely access to local sexual health services and treatment after accessing HIV self-testing would be necessary for this intervention’s successful implementation. They further highlighted how receiving a positive self-test result in isolation could lead to self-harm and suicide ideation, especially if the individual does not have social or emotional support networks available. Douglas (20s, *cis* man) contemplated the harm that may be associated with HIV self-testing in relation to HIV stigma, diagnosis, and emotional distress:

There’s still stigma with HIV and there’s still a high suicide rate with HIV folks. And there I feel like there’s a risk if someone does test positive at home and they don’t have support. (Service User Focus Group)

Yet at the same time, many providers emphasized that instead of withholding HIV self-testing out of fear for potential misuse or harm, providers should ‘trust clients or patients or people who want to get tested to make decisions that are good for them’ (Landon, Sexual Health Nurse, Service Provider Interview). Kendall, a front-line worker, made an apt comparison with another popular at-home test:

People were very concerned about home pregnancy tests when they came on the market and they’re fine […] I don’t have actual huge concerns that people are going to do their tests wrong, or they’re going to misuse them, or that they’re going to do a test that they’re really not prepared to get an answer on. I think people are pretty good at making those calls for themselves. (Service Provider Interview)

Ultimately, both 2SGBQM and providers believed that HIV self-testing may be more suitable for regular or experienced testers who conduct routine HIV testing. For first-time or inexperienced testers, participants believed that they should meet with a provider first to receive appropriate sexual health education and information on the available supports and resources as a way to reduce the potential risks and harm that may be associated with HIV self-testing.

## Discussion

Overall, both providers and 2SGBQM supported the implementation and adoption of finger-prick HIV self-tests, and most 2SGBQM reported that they would access HIV self-testing once it became available, demonstrating the intervention’s high acceptability. These findings support previous results from both Canadian (Pant Pai *et al*. 2014, 2018a, 2018b, O'Byrne *et al*. 2021a, 2021b, 2023a, 2023b) and international (Witzel *et al*. 2020, Jamil *et al*. 2021) HIV self-testing literature which demonstrate that HIV self-testing is acceptable among a wide range of populations, including providers and priority population groups like 2SGBQM. The high acceptability of HIV self-testing among our participants was due to its perceived benefits, such as convenience (i.e. less time and effort), removal of social and structural barriers to testing, empowerment of service users, and reduced workload for providers. Earlier studies have similarly shown that sexual health service users find HIV self-testing much more convenient, accessible, confidential, and empowering than clinic-based testing, and that they prioritize convenience and privacy within their testing decision-making process (Pant Pai *et al*. 2014, 2018a, Witzel *et al*. 2020, Jamil *et al*. 2021).

Our participants shared the belief that the benefits of HIV self-testing may encourage individuals who never or rarely get tested for HIV using traditional clinic-based services, and that HIV self-testing could be the ‘gateway’ experience leading to regular testing, timely treatment, and further sexual health education. Results from Canadian studies such as GetaKit corroborate this belief, illustrating that HIV self-testing can indeed increase access for individuals who have not previously tested for HIV, especially for those who belong to priority population groups (O'Byrne *et al*. 2021a, 2021b, 2023a, 2023b). Among participants (*n* = 398) who accessed HIV self-testing through the GetaKit study between July 2020 and April 2021 in Ottawa, Ontario, a quarter (23.9%) had no prior HIV testing experience, and most participants—and around half of all first-time testers in the study—were part of at least one priority population group (O'Byrne *et al*. 2021b).

While most of our 2SGBQM participants reported that they would access HIV self-testing once it became available, some explained that their uptake of this intervention would require weighing its perceived benefits and concerns, and depend on the situational context at the time of seeking testing. Reasons for hesitancy included fear of needles/blood and the belief that HIV self-tests may not be as accurate or reliable as clinic-based blood-draw tests. Yet many studies have demonstrated the high accuracy and reliability of HIV self-testing in a wide range of contexts (Witzel *et al*. 2020, Galli *et al*. 2021, Jamil *et al*. 2021, Singh *et al*. 2023); in the Canadian context, the INSTI finger-prick HIV self-test has reported sensitivity of 99.8% and specificity of 99.5% (Galli *et al*. 2021, bioLytical Labratories 2025a, 2025b, 2025c). This dissonance between the high accuracy of HIV self-tests and our 2SGBQM participants’ perception of low accuracy may be due to the fact that our interviews and focus groups with the majority of our 2SGBQM took place prior to the approval of HIV self-testing in Canada. All but one of our 2SGBQM participants had heard of HIV self-testing prior to participation, and the lack of exposure to and awareness of HIV self-testing may have led to lower levels of trust in this testing modality. Our results therefore illustrate the importance of educating sexual health service users about the reported accuracy and reliability of HIV self-tests, which would increase trust in this intervention, and in turn, its acceptability and uptake. It is equally important to educate those accessing HIV self-testing about the window period of 3–12 weeks postexposure during which HIV antibodies typically become detectable, so that testers understand when re-testing or confirmatory testing is recommended to maintain an accurate assessment of their HIV status. Opportunities for such sexual health education include clinic-based testing encounters where service providers can share information and resources during casual conversation, targeted peer-led outreach at community events and social venues frequented by 2SGBQM, online resources (i.e. articles, tutorials, or webinars), in-app advertising or social-media messaging through platforms frequented by 2SGBQM, and integration of HIV self-testing education into existing pre-exposure prophylaxis (PrEP) and STBBI testing programmes. Other HIV self-testing modalities, such as saliva-based testing, may also increase the acceptability and uptake of HIV self-testing by removing the fear of needles/blood as a major barrier to accessing this intervention.

2SGBQM and providers were also concerned that some self-testers may experience extreme levels of emotional distress when receiving a positive result due to existing HIV stigma, especially if they have limited knowledge of HIV or the available supports, resources, and treatments. Participants suggested that in-person services may be best suited for those who are inexperienced with HIV testing, or less informed about HIV in general. However, systematic reviews of HIV self-testing literature have found that there were no incidents of self-harm or suicide as a result of accessing HIV self-testing among the 24 reviewed studies, and that there were similar proportion of people who were diagnosed and linked to care through HIV self-testing as with clinic-based testing (Witzel *et al*. 2020, Jamil *et al*. 2021). These findings support our providers’ belief that sexual health clients can indeed be trusted to manage their own sexual health needs after receiving a positive diagnosis, such as seeking treatment or emotional support. Nonetheless, offering HIV self-testing in conjunction with accessible clinic-based testing and other in-person services such as counselling are essential in meeting the diverse needs of individuals and communities seeking HIV testing, while also reducing the risks of potential harms that may arise from accessing HIV self-testing. Furthermore, as reflected in our previous contributions, persistent HIV stigma within healthcare settings continues to represent critical gaps within the sexual healthcare continuum, and there is a critical need for ongoing efforts to reduce HIV-related stigma through system-wide stakeholder engagement, education, and structural change (Grace *et al*. 2022, Stewart *et al*. 2022, Dulai *et al*. 2023, 2024, Hassan *et al*. 2025).

Finally, while our participants believed that HIV self-testing should be available for free, they also suggested that up to $20 CAD would be an acceptable price for HIV self-tests, similar to the findings of earlier Canadian studies (Pant Pai *et al*. 2014, 2018a, 2018b). However, the current cost of purchasing HIV self-testing kits ($34.95 CAD when purchased from the manufacturer) is almost twice the acceptable price identified by our participants (bioLytical Labratories 2025c). In addition, the one-time federal funding for wide and affordable distribution of HIV self-testing has ended without renewal in sight (CBC News 2024, Malone 2024). This is despite the success of HIV self-testing programmes such as GetaKit in enabling access to HIV testing for members of priority populations (O'Byrne *et al*. 2021a, 2021b, 2023a, 2023b), and the ability of alternative sexual health testing modalities like HIV self-testing in strengthening the resilience of our healthcare system during times of disruptions (O'Byrne *et al*. 2021a, Ryu *et al*. 2023).

In light of the lack of federal support for HIV self-testing, local public health units and community-based organizations may collaborate to secure bulk purchasing agreements and reduce the per-unit cost of acquiring HIV self-tests, or strategically integrate HIV self-testing into existing PrEP, STBBI testing, and community outreach programmes by dedicating a portion of their budget towards the distribution of HIV self-tests. Since sustained and equitable access to HIV testing and care depend on coordinated public investment, but vary across provincial boundaries and healthcare systems, we hope that the clear, cross-stakeholder demand for HIV self-testing and its individual and system-level benefits evidenced by our research can support the advocacy for its renewed national funding across Canada. The grounded perspectives and detailed insights in our research may also guide further efforts in the monitoring and evaluation of HIV self-testing interventions, and inform which outcomes warrant attention as they are most meaningful, salient, and relevant to both providers and 2SGBQM.

While testing is a crucial first step within the HIV care continuum, meaningful progress towards the Pan-Canadian STBBI Framework for Action or the UNAIDS 95-95-95 targets cannot be made without continued investment across the full spectrum of sexual healthcare, including repeat testing, counselling, PrEP, and ART. If Canada is truly committed to reducing future transmissions of HIV, it is imperative that HIV self-testing and the broader spectrum of sexual health services are made universally accessible across diverse communities through national funding and a coordinated sexual health strategy.

### Strengths and limitations

One of the greatest strengths of our study is the use of CBPR principles and RTA techniques. The use of CBPR principles increases the credibility and validity of our findings by meaningfully involving 2SGBQM in all stages of research as both peer researchers and CAB members, grounding the produced knowledge within the lived realities of the community being studied. The use of RTA techniques also adds rigour and validity as peer researchers engaged deliberately and conscientiously with the data during analysis. Another strength of our research is the incorporation of varied experiences and perspectives of providers and 2SGBQM differing in age, ethnicity, sexual orientation, gender identity, work experience, and geographical location across Ontario. Collecting data from both 2SGBQM and providers allowed us to procure a wide range of data and create a comprehensive understanding of factors influencing the acceptability and uptake of HIV self-testing. The use of virtual focus groups and interviews as study instruments contributed to the diversity of our participants and data by allowing participation with fewer time constraints and regardless of geographic location.

At the same time, there are some limitations to our study. First, while our sample captures a diverse array of providers and 2SGBQM, most were based in urban regions. Recruitment of more participants from rural and remote regions may have highlighted different insights and outcomes regarding HIV self-testing acceptability and uptake. The virtual nature of our focus groups and interviews may also have limited participation for those who do not have adequate access to the internet or required technology. And since our research was only conducted in English, individuals with no or low English proficiency may have been excluded from our study. Future research employing multiple languages, offline recruitment, and in-person data collection would be crucial in addressing such gaps and gaining further insights into HIV self-testing acceptability and uptake among a broader range of 2SGBQM. Additionally, focus groups and interviews with those who have never or not recently accessed STBBI testing may provide different insights than those included in our study. However, our study is unique in that data collection began prior to the approval of HIV self-testing in Canada and continued through its early implementation. This timing of our research enabled us to capture early perspectives of 2SGBQM and providers who have not yet been exposed to or influenced by widespread public health campaigns or media coverage advocating the intervention’s potential positive impacts and benefits, instead illustrating how foundational beliefs, expectations, and individual- and system-level considerations shaped early interest in, and barriers to HIVST uptake and implementation.

## Conclusion

Our findings suggest that finger-prick HIV self-testing is acceptable to sexual health service providers and 2SGBQM service users in Ontario, Canada. While participants identified many potential benefits of HIV self-testing, some 2SGBQM were hesitant to access HIV self-testing. Both providers and 2SGBQM believed that traditional clinic-based testing may be best suited for first-time or infrequent testers, while HIV self-testing could empower experienced or regular testers to take control of their own sexual health. At the same time, both groups also believed that HIV self-testing may be a gateway to regular testing, sexual health education, and connections to care for those who never or rarely get tested for HIV through clinic-based testing. HIV self-testing is an alternative testing modality that could help diagnose the thousands of individuals living in Canada who are unaware of their sero-positivity, connect them to the HIV care continuum, and lead to timely treatment, improved sexual health outcomes, viral suppression, and reduced transmission of HIV. With the high cost of accessing HIV self-testing in Canada and the lack of national public funding for its wide distribution, it is important to recognize the positive impact that HIV self-testing can have, and find ways to ensure that alternative sexual health testing modalities like HIV self-testing can be accessible for everyone.

## Data Availability

All relevant data are within the article, and the data underlying this article cannot be shared publicly due concerns for participant anonymity.
